# Supplementary material for: Néel spin-orbit torque in antiferromagnetic quantum spin and anomalous Hall insulators
Source: Nat Commun. 2025 Aug 21;16:7790. doi: 10.1038/s41467-025-63171-1 (PMC12370977; doi:10.1038/s41467-025-63171-1)
Supplement: Supplementary file 1 — Supplementary Information [file 41467_2025_63171_MOESM1_ESM.pdf]

Supplemental Information for “Néel Spin-Orbit Torque in Antiferromagnetic Quantum Spin and Anomalous Hall Insulators"

Junyu Tang,[1] Hantao Zhang,[2] and Ran Cheng[2,1,3]

[1]*Department of Physics and Astronomy, University of California, Riverside, California 92521, USA*

[2]*Department of Electrical and Computer Engineering, University of California, Riverside, California 92521, USA*

[3]*Department of Materials Science and Engineering, University of California, Riverside, California 92521, USA*

CONTENTS

I. Topological phases 2

II. Non-equilibrium spin generation 3

III. Antiferromagnetic resonance 5

I. Topological phases

To provide further information about the the topological electrons, we plot additional phase diagrams different from the main text. As a benchmark, we first plot the phase diagram for the original Kan-Mele model excluding the collinear AFM background order in Fig. S1(a). In the central region with small λ_v and λ_R, the system is in the QSH state with a Z_2 number equals $+1$. After introducing a collinear AFM background order $\boldsymbol{m}_A = -\boldsymbol{m}_B = +\hat{z}$, the Z_2 number is no longer well defined as the time reversal symmetry is broken. Instead, we plot the Chern number C in Fig. S1(b) with the same parameters as Fig. S1(a). The central QSH region remains but with a shrunk size. Two QAH regions open up, with opposite Chern numbers, separating the central QSH region from the NI regions.

In Fig. S1(c), we plot the Chern number C as a function of λ_{ex} and λ_{soc}. The QAH phases exist only when the exchange coupling is nonzero ($\lambda_{ex} \neq 0$). By comparing Figs. S1(a–c) with Figs. 2(a–d) of the main text, we find that, once the exchange coupling is included, a direct phase transition from NI to QSH is prohibited while the system must pass through the QAH phase.

In the QSH phase, the Chern number C is zero, so we have to resort to the spin Chern number C_s and the band structure, as discussed in the main text. Here we provide an additional evidence from a real-space perspective. We truncate our periodic Hamiltonian in the y direction with a width of $N = 40$ unit cells and in the x direction with $N = 80$ unit cells to obtain a scattering region, so the system becomes finite in both directions. We then calculate the scattering states from the left and right leads with an energy fixed in the bulk gap. Figs. S2(a) and (b) show the spin-resolved distribution of the scattering states, where the right-propagating (left-propagating) current mainly

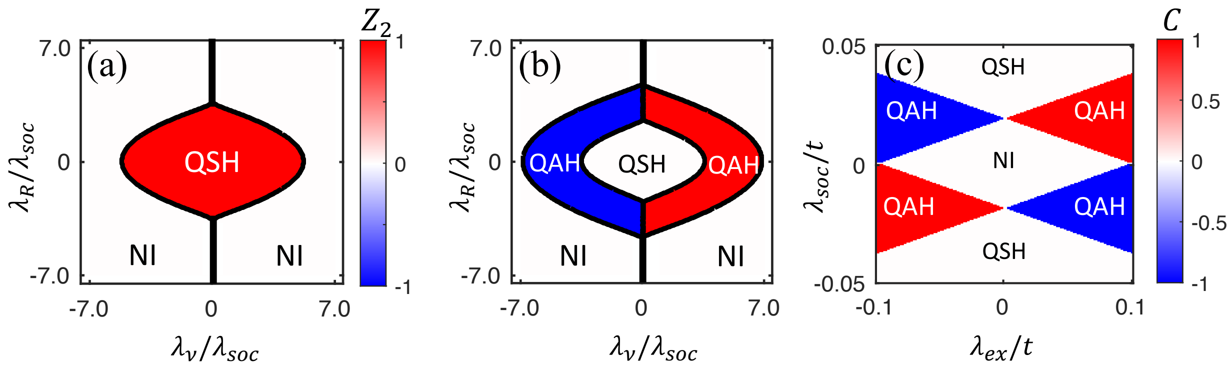

FIG. S1. Phase diagrams of (a) Z_2 number in the absence of the AFM order (non-magnetic Kane-Mele model) for $\lambda_{soc} = 0.06t$ and $\lambda_{ex} = 0$; (b) Chern number in the presence of the AFM order for $\lambda_{soc} = 0.06t$ and $\lambda_{ex} = 0.1t$; and (c) Chern number for $\lambda_v = \lambda_R = 0.05t$.

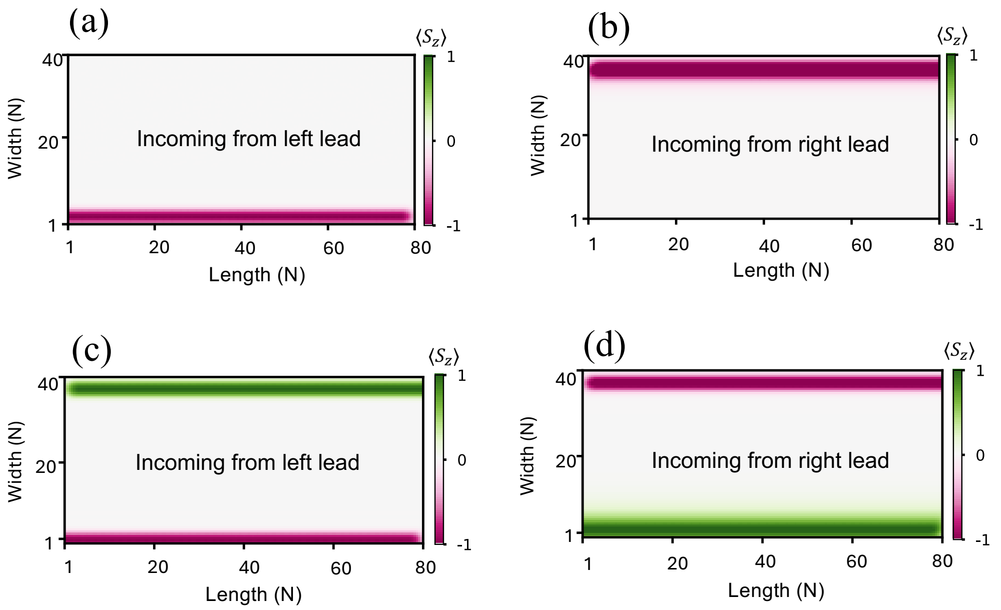

FIG. S2. Spatial distribution of the normalized spin-resolved edge electron transport in a finite sample (width×length = 40×80) for the QAH state with $\lambda_{soc} = 0.02t$ [(a) and (b)] and the QSH state with $\lambda_{soc} = 0.05t$ [(c) and (d)]. In both cases, we set $\lambda_R = 0.025t$, $\lambda_v = 0.05t$, and $\lambda_{ex} = 0.1t$.

locates at the lower (upper) edge. Additionally, these scattering states both have a spin-down polarization, consistent with the physical picture of the QAH effect. As λ_{soc} is further increased to $0.05t$, the corresponding spin-resolved distribution of the scattering states are plotted in Figs. S2(c) and (d). Distinct from the QAH case, here both the right- and left-propagating currents appear at each edge. Moreover, the states on opposite edges exhibit opposite spin polarization, consistent with the physical picture of the QSH effect.

II. Non-equilibrium spin generation

The finite staggered potential λ_v breaks the $\mathcal{PT}$-symmetry, enabling finite nonzero Berry curvature. To further investigate the relation between the non-equilibrium spin accumulation $\delta\boldsymbol{S}^{A,B}$ and the band topology, we turn on λ_v to ensure the presence of the QAH phase. However, a nonzero λ_v would render $|\delta\boldsymbol{S}^A| \neq |\delta\boldsymbol{S}^B|$ owing to the sublattice imbalance, which leads to a finite $\delta\boldsymbol{S} = (\delta\boldsymbol{S}^A + \delta\boldsymbol{S}^B)/2$ on top of $\delta\boldsymbol{N} = (\delta\boldsymbol{S}^A - \delta\boldsymbol{S}^B)/2$, hence inducing a nonzero total SOT besides the NSOT. This can be seen in Fig.4(b) of the main text where, as $|\lambda_v|$ increases, the relation $\delta\boldsymbol{S}^A = -\delta\boldsymbol{S}^B$ becomes increasingly inaccurate.

Here, with a finite $\lambda_v = 0.05t$, we plot $\delta\boldsymbol{S}^A$ and $\delta\boldsymbol{S}^B$ as functions as λ_R and λ_{ex} in Figs. S3(a)

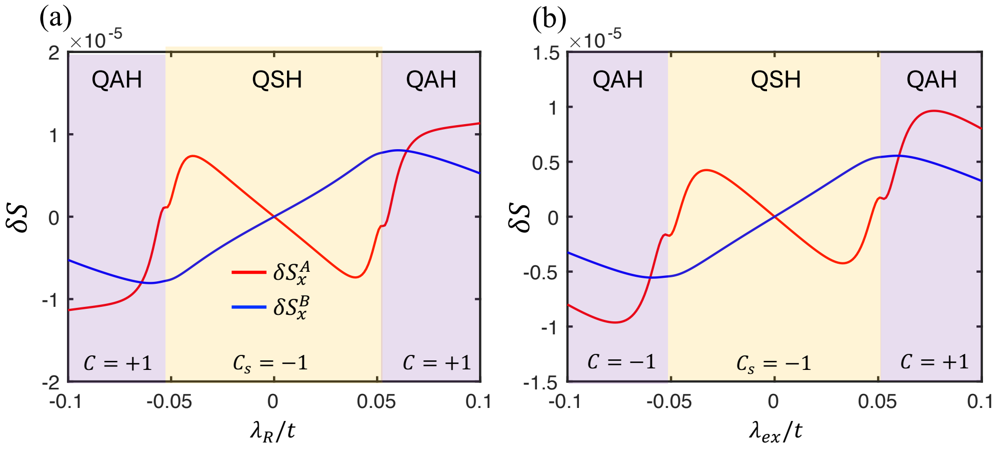

FIG. S3. Non-equilibrium spin accumulation per unit cell (in units of $\hbar/2$) for each sublattice as a function of (a) Rashba SOC λ_R and (b) exchange interaction γ_{ex}. In (a), we set $\lambda_{soc} = 0.04t$, $\lambda_v = 0.05t$, $\lambda_{ex} = 0.1t$. In (b) we set $\lambda_R = \lambda_v = 0.05t$, $\lambda_{soc} = 0.03t$. In both figures, we adopt $E_x = 1$ V/μm, $t = 1$ eV.

and (b). They exhibit a similar pattern, and the QAH-to-QSH transition is smoothened by a finite broadening factor $\Gamma = 20$ meV to avoid divergence at the band closing points. Here, we can also find that the NSOT feature ($\delta \boldsymbol{S}^A$ and $\delta \boldsymbol{S}^B$ have opposite signs) is most robust in the QSH state. In Figs. S4(a) and (b), we also plot the angular dependence for $\delta \boldsymbol{S}$. Note that the NSOT studied in our work is carried by the bulk adiabatic currents, which should not be confused with the edge currents discussed above.

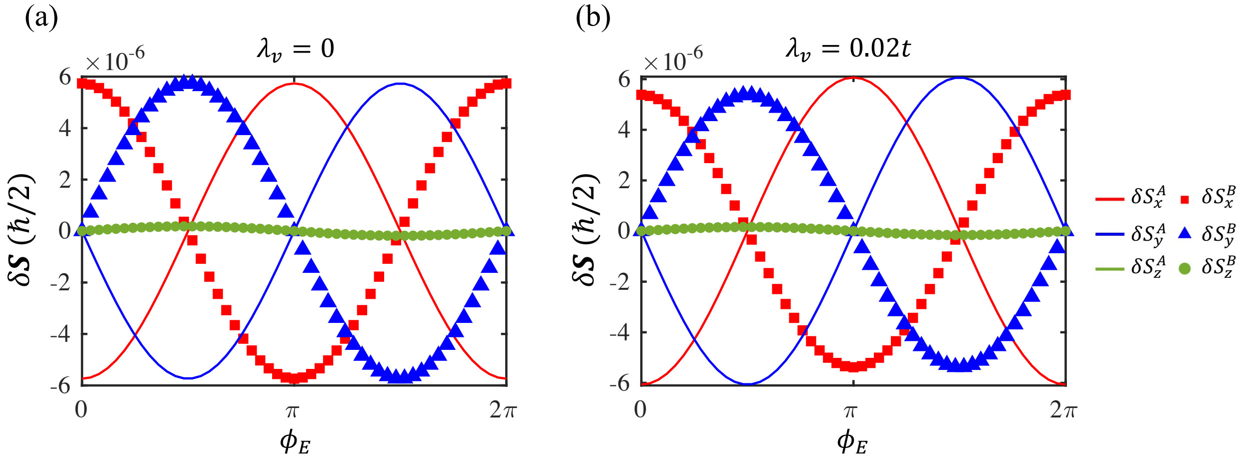

FIG. S4. The non-equilibrium spin accumulation $\delta \boldsymbol{S}$ per square nanometer as a function of the in-plane azimuthal angle ϕ_E of the applied electric field when the staggered potential λ_v is (a) turned off; and (b) turned on. Parameters : $\lambda_R = \lambda_{soc} = 0.05$, $\Gamma = 20$ meV, and $E_x = 1$ V/μm.

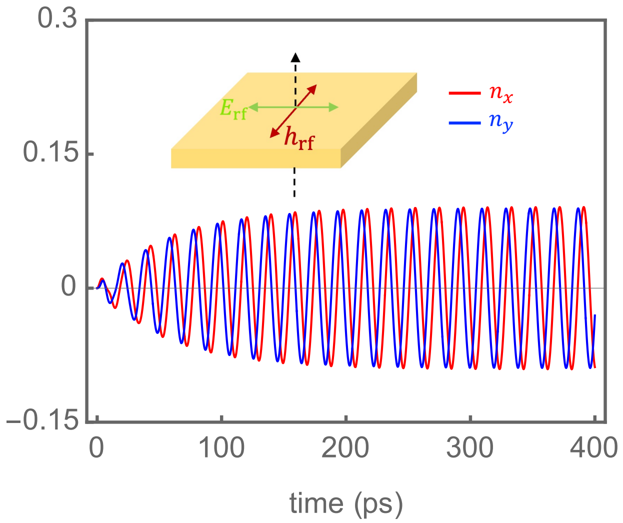

FIG. S5. Time evolution of the Néel order $\boldsymbol{n}$ with the onset of AFM resonance (frequency: $f_r = 51.9$ GHz) for an normally incident electromagnetic wave (inset). Parameters: $H_J = 35$ T, $H_\parallel = 0.16$ T, $H_0 = 1.5$ T, $\alpha = 0.005$, $E_x = 0.5\mathrm{V}/\mu\mathrm{m}$, and $\Gamma = 20$ meV

III. Antiferromagnetic resonance

In the main text, we have compared the two cases where the AFM resonance is directly driven by either h_{rf} or the NSOT-induced h_{NS} originating from the same electromagnetic wave. Here, we provide a third case where the Poynting vector of the electromagnetic wave is perpendicular to the sample plane (see the inset in Fig. S5). In this scenario, both the electric component and the magnetic component can drive the AFM resonance. We plot the time evolution of the Néel order with the onset of AFM resonance in Fig. S5. The results are almost the same as Fig.5(b) in the main text. This demonstrates the negligible effects of the uniform SOT comparing with our NSOT when $|h_{rf}| \approx |h_{NS}|$.
